# Pre-conceptional Maternal Vitamin B12 Supplementation Improves Offspring Neurodevelopment at 2 Years of Age: PRIYA Trial

**DOI:** 10.3389/fped.2021.755977

**Published:** 2021-12-07

**Authors:** Naomi D'souza, Rishikesh V. Behere, Bindu Patni, Madhavi Deshpande, Dattatray Bhat, Aboli Bhalerao, Swapnali Sonawane, Rohan Shah, Rasika Ladkat, Pallavi Yajnik, Souvik K. Bandyopadhyay, Kalyanaraman Kumaran, Caroline Fall, Chittaranjan S. Yajnik

**Affiliations:** ^1^Diabetes Unit, King Edward Memorial Hospital Research Center, Pune, India; ^2^Terre des Hommes Rehabilitation and Morris Child Development Centre at KEM Hospital, Pune, India; ^3^Strategic Consulting, Cytel Inc., Cambridge, MA, United States; ^4^Medical Research Council Lifecourse Epidemiology Unit, University of Southampton, Southampton, United Kingdom

**Keywords:** vitamin B12, pre-conception, supplementation, neurodevelopmental outcome, offspring

## Abstract

**Background:** The first thousand days window does not include the pre-conceptional period. Maternal pre-conceptional health has a profound influence on early embryonic development (implantation, gastrulation, placentation etc). Nutrition provided by B-complex vitamins is important for fetal growth, especially neural development. We report effects of a maternal pre-conceptional vitamin B12 and multi micronutrient (MMN) supplementation on offspring neurodevelopmental performance.

**Methods:** In the Pune Rural Intervention in Young Adolescents trial (PRIYA), adolescents (*N* = 557, 266 females) were provided with vitamin B12 (2 μg/day) with or without multiple micronutrients, or a placebo, from preconception until delivery. All groups received mandatory iron and folic acid. We used the Bayley's Scale of Infant Development (BSID-III) at 24–42 months of age to investigate effects on offspring neurodevelopment.

**Results:** Participants had similar baseline B12 levels. The levels improved in the B12 supplemented groups during pre-conception and pregnancy (28 weeks gestation), and were reflected in higher cord blood holotranscobalamin (holo-TC) levels compared to the placebo group. Neurodevelopmental outcomes in the B12 alone group (*n* = 21) were better than the placebo (*n* = 27) in cognition (*p* = 0.044) and language (*p* = 0.020) domains (adjusted for maternal baseline B12 levels). There was no difference in neurodevelopmental outcomes between the B12 + MMN (*n* = 26) and placebo group. Cord blood Brain Derived Neurotrophic Factor (BDNF) levels were highest in the B12 alone group, though not significant.

**Conclusion:** Pre-conceptional vitamin B12 supplementation improved maternal B12 status and offspring neurodevelopment at 2 years of age. The usefulness of cord BDNF as a marker of brain development needs further investigation. Our results highlight the importance of intervening during pre-conception.

## Introduction

The developing fetus is dependent on its mother for its nutrition. Maternal nutrition before and during pregnancy affects fetal growth and development, and maternal malnutrition may predispose the offspring to undesirable outcomes in later life. This concept is called “fetal programming.” This is the backbone for the Developmental Origins of Health and Disease (DOHaD) paradigm which expanded the idea to include “health” as a programmed state (1, 2). Pregnancy and the first 2 years of life (1,000 days) are considered the most crucial window for programming (3).

Maternal nutritional factors (both macro and micronutrients) influence neurodevelopmental processes *in utero*, such as neurogenesis, myelination, synaptogenesis, and cortical brain growth (3).Vitamins B12 and folate are of special interest due to their role in the one carbon metabolism pathway. This represents a series of biochemical reactions involving the methionine and folate cycles. The methylation of homocysteine involves the transfer of a methyl group from 5-methyl tetra-hydro folate (THF) by methionine synthase (MS). Vitamin B12 is a cofactor for this reaction. This transfer in turn generates S-adenosyl methionine (SAM) which is a universal methyl donor. One carbon metabolism supports important cellular processes such as DNA synthesis, repair, and methylation, which is important for epigenetic regulation of gene expression (4). Offspring of mothers with low maternal vitamin B12 and folate status during pregnancy have a higher risk of neural tube defects and neurodevelopmental disorders [Autism, Attention Deficit Hyperactivity Disorder (ADHD)], poorer cognitive development, and smaller brain volumes in childhood (5–8). In animal models (rats), offspring of mothers exposed to a high folate and low vitamin B12 diet show lower levels of Brain Derived Neurotrophic Factor (BDNF) in the brain, and poorer cognitive function (9, 10).

In India, vitamin B12 deficiency is widely prevalent in pregnant women (50–70%) (11, 12) and is attributable to the socio-cultural practice of vegetarianism and poor economic status (13–16). This deficiency is associated with a range of adverse pregnancy and offspring health outcomes (17). In prospective birth cohorts from western India, we have earlier shown that exposure to low maternal vitamin B12 *in utero* is associated with poorer cognitive functioning at the age of 2 and 9 years in the offspring (18, 19). However, public health policy in India mandates only iron and folic acid supplementation to women in the reproductive age group, and during pregnancy and lactation. A randomized controlled trial in South India showed that supplementing 50 μg/day oral B12 from 14 weeks of pregnancy until 6 weeks postpartum improved B12 concentrations in breast milk, the vitamin B12 status of infants at 6 weeks and infant cognitive function at 30 months of age (20, 21).

Important milestones in fetal neural development such as neural tube closure are completed by 26–28 days of gestation (22). The majority of pregnancies in India are unplanned, and by the time pregnancy is detected (typically between 10 and 14 weeks gestation) this early developmental window is lost. Pre-conceptional supplementation will ensure that the mother has improved vitamin stores during the early neurodevelopmental period. The success of pre-conceptional folic acid supplementation in preventing neural tube defects is well-known (23–25). Few studies have examined the effects of pre-conceptional maternal micronutrient supplementation on offspring neurodevelopment in India. This approach will expand the 1,000 days concept to include the pre-conceptional period.

The Pune Rural Intervention in Young Adolescents (PRIYA) is a pre-conceptional vitamin B12 and multi micronutrient supplementation trial in adolescent participants of the Pune Maternal Nutrition Study. Here we report neurodevelopmental outcomes at 2 years of age in the offspring of female participants in the trial. We hypothesized that pre-conceptional B12 supplementation in the mothers would contribute to better neurodevelopmental outcomes in their offspring.

## Materials and Methods

### PRIYA Trial

**Figure 1:**
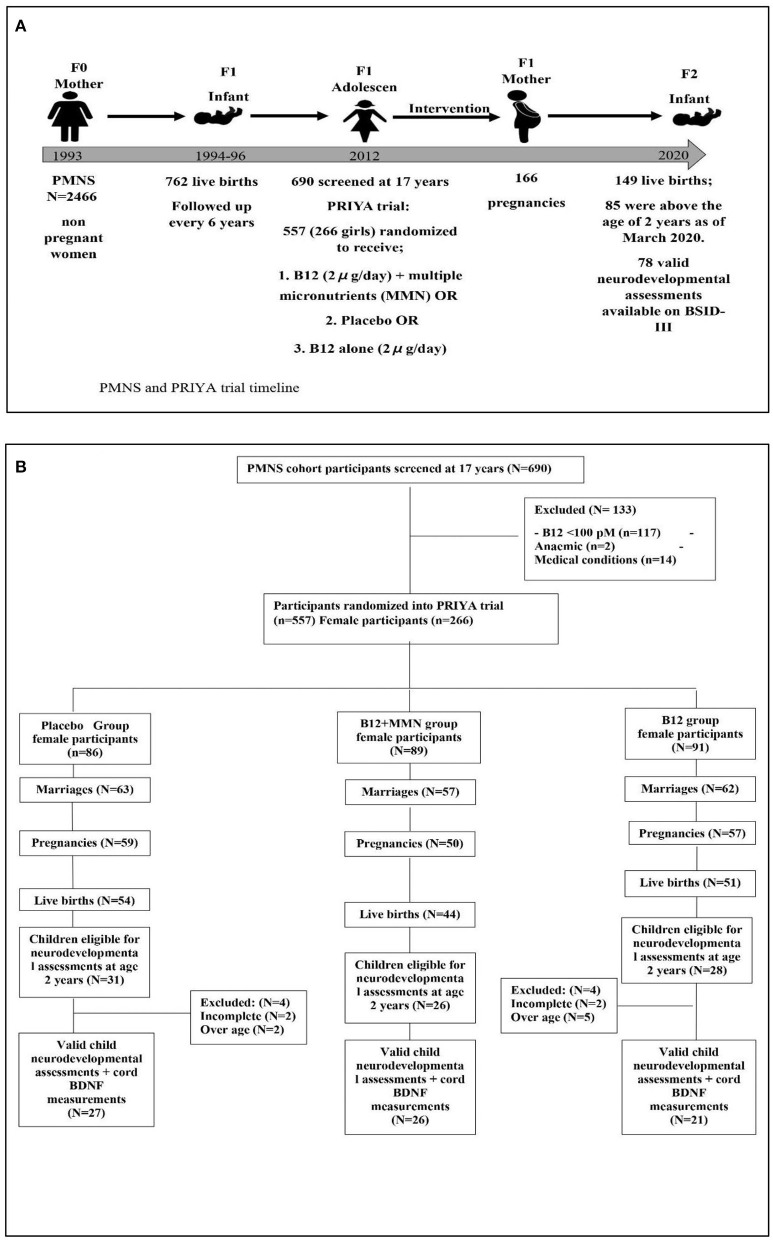
Diagram depicting the study timeline **(A)** and recruitment of study participants **(B)** for neurodevelopmental follow up as of February 2020. Further collection of data discontinued due to COVID-19 pandemic.

The PRIYA trial methods have been published previously (26). Briefly, The Pune Maternal Nutrition Study (PMNS) is a pre-conceptional observational birth cohort set up in 1993 (Figure 1). Married non-pregnant women were recruited from six villages around Pune and those who became pregnant were followed up. Seven hundred and sixty-two children were born and followed up serially. At ~17 years of age, 690 participants from the PMNS cohort (11) were screened for inclusion in the PRIYA trial. Of these, 117 were excluded due to severe vitamin B12 deficiency (<100 pmol/L) because of the ethical imperative of a placebo-controlled trial. Sixteen were excluded due to systemic illnesses. Five hundred and fifty-seven (266 females) participants were randomized (Figure 1) to receive either a placebo, B12 (2 μg/day) + multiple micronutrients (MMN) or B12 alone (2 μg/day). The composition of the MMN tablet (Supplementary Table 1) was guided by the WHO/UNICEF/UNU international multiple micronutrient preparation (UNIMMAP). We excluded Iron and Folic Acid because the mandated IFA tablets (Iron and Folic Acid) were given to all participants as per Government of India recommendations (100 mg elemental Iron and 500 μg folic acid once a week during adolescence, and at least 100 tablets during pregnancy). The investigational supplements (vitamin B12 containing) were continued for the female participants daily until their first delivery. They and the study team were blinded to the vitamin/micronutrient supplementation.

This paper describes the findings in the female participants and their children. Participants were followed up regularly for health problems, and marriages were recorded. Married women were monitored to detect pregnancy which was confirmed by a urine pregnancy test. At 24–28 weeks gestation, mothers visited the Diabetes Unit, KEM Hospital Research Center Pune, for a fasting oral glucose tolerance test (as per international guidelines) and clinical and biochemical evaluations. The clinical evaluation included anthropometric measurements, an obstetric consultation and estimate of fetal growth by ultra-sonography. We also obtained socio demographic information (assessed using the Standard of Living Index questionnaire from the National Family Health Survey of India (NFHS).

Details of deliveries were recorded (gestational age and type of delivery). Cord blood samples were collected and processed for hematological and nutrient measurements. We performed detailed anthropometric measurements on the baby within 72 h of birth.

We measured circulating concentrations of vitamins B12, holotranscobalamin (holo-TC), folate, and total homocysteine at baseline, 6–12 months after the start of the supplementation (at ~18 years of age), 28 weeks gestation, and in cord blood (Table 1). We additionally measured B2 and B6 levels in mothers at 28 weeks gestation and in offspring cord blood.

**Table 1:** Maternal characteristics at baseline and in pregnancy, and child characteristics.

**Variables**	***n* ^#^**	**Placebo group**	***n***	**B12 + MMN group**	***n***	**B12 group**	***p*-values**	
**Parental sociodemographic characteristics**								
Maternal age at 28 weeks gestation (years)	25	19.8 (1.0)	25	19.4 (1.1)	21	19.7 (1.1)	0.555	
Maternal education (years)	26	12.5 (11.0, 13.0)	26	12.0 (10.0, 13.0)	21	12.0 (11.0, 13.5)	0.639	
Maternal height (cms)	25	158.2 (5.2)	25	158.7 (5.0)	21	157.8 (4.8)	0.852	
Maternal weight at 28 weeks gestation (kgs)	25	55.4 (48.6, 59.3)	25	51.2 (49.4, 54.4)	21	52.9 (47.0, 60.7)	0.656	
Maternal IQ	21	76.6 (9.5)	15	74.4 (8.8)	17	75.8 (7.2)	0.751	
Standard of Living Index	26	36.0 (30.5, 40.5)	26	38.0 (31.0, 40.0)	21	37.0 (32.0, 40.0)	0.923	
Paternal Education (years)	25	14.0 (10.5, 15.0)	24	12.0 (10.0, 15.0)	19	12.0 (10.0, 15.0)	0.656	
Duration of supplementation (months)	27	34.0 (22.0, 45.0)	26	36.0 (21.0, 45.2)	21	35.0 (23.0, 49.5)	0.937	
Compliance (percentage)	27	89.4 (76.0, 94.8)	26	77.5 (67.8, 88.5)	21	81.5 (72.3, 87.8)	0.649	
**Maternal micronutrients**							***p*****-values (B12** **+** **MMN vs. Placebo)**	***p*** **-values (B12 vs. Placebo)**
**At screening**								
Vitamin B12 (pM)	27	151.0 (122.0, 193.0)	26	159.5 (134.0, 219.0)	21	138.0 (125.0, 190.0)	0.350	0.860
Holo-TC	27	11.0 (8.3, 13.4)	26	11.1 (6.2, 15.8)	21	7.9 (5.3, 11.4)	0.87	0.30
Folate (nM)	27	20.9 (15.3, 24.6)	26	15.7 (11.3, 26.6)	21	20.8 (15.3, 29.1)	0.357	0.698
Homocysteine (μmol/L)	27	20.1 (15.1, 38.0)	26	18.6 (15.3, 30.3)	21	27.5 (17.0, 39.6)	0.646	0.434
**At 18 years**								
Vitamin B12 (pM)	25	162.0 (125.9, 192.5)	26	285.0 (205.8, 368.7)	18	274.7 (224.7, 388.2)	<0.001***	<0.001***
Holo-TC	27	9.5 (6.2, 14.9)	26	22.6 (8.7, 29.2)	19	25.2 (8.1, 36.0)	<0.001***	<0.001***
Folate (nM)	26	23.0 (17.2, 29.8)	26	21.2 (15.3, 28.8)	18	20.4 (14.9, 28.3)	0.734	0.925
Homocysteine (μmol/L)	27	16.7 (11.7, 28.3)	26	9.60 (8.30, 13.4)	18	10.6 (9.22, 16.0)	<0.001***	0.013*
**At 28 weeks gestation**								
Hemoglobin (gm/dl)	25	10.4 (9.5, 11.0)	25	10.2 (9.4, 11.0)	21	10.4 (9.1, 10.7)	0.491	0.638
Vitamin B12 (pM)	25	134.0 (95.5, 163.0)	25	164.0 (149.0, 218.5)	21	204.0 (173.5, 261.0)	0.007**	<0.001***
Holo-TC (pM)	25	14.8 (8.85, 25.1)	25	21.9 (15.3, 36.5)	21	21.3 (16.9, 36.8)	0.027*	0.012*
Folate (nM)	25	47.9 (18.0, 71.5)	25	20.6 (10.2, 49.7)	21	28.5 (16.6, 51.4)	0.043*	0.302
Vitamin B2 (pM)	25	244.0 (210.5, 273.0)	25	276.0 (229.5, 304.5)	20	244.0 (221.7, 269.5)	0.028*	0.852
Viamin B6-pyridoxal-5-phospate (pM)	24	3.5 (2.3, 4.6)	25	4.6 (3.3, 7.4)	21	3.1 (2.6, 4.8)	0.117	0.357
Vitamin B6-pyridoxal (pM)	15	1.0 (0.8, 1.6)	12	1.1 (0.9, 1.3)	12	1.3 (1.0, 1.7)	0.786	0.922
Homocysteine (μmol/L)	25	7.0 (5.0, 9.2)	25	6.3 (4.3, 8.1)	21	5.1 (3.9, 7.2)	0.559	0.550
**Child characteristics**								
Child age at assessment (months)	27	27 (26, 34)	26	29 (27, 36.2)	21	29 (26, 32)	0.623	0.901
Gender	27	Boys = 18 (66.7%)	26	Boys = 13 (50%)	21	Boys = 11 (52.3%)		
**Birth anthropometry**								
Gestation age (weeks)	27	39.0 (38.0, 40.2)	26	39.0 (38.0, 40.2)	21	39.4 (38.8, 40.2)	0.920	0.936
Birth weight (gm)	27	2,908.6 (412.5)	26	2,809.2 (458.6)	21	2,788.9 (315.9)	0.411	0.277
Birth length (cm)	27	49.1 (46.8, 49.8)	26	48.2 (47.4, 49.8)	20	48.5 (47.2, 49.3)	0.990	0.328
Head circumference (cm)	27	33.4 (1.0)	26	33.1 (1.0)	20	33.0 (0.9)	0.237	0.142
**Cord micronutrients**								
Vitamin B12 (pM)	27	226.0 (138.0, 289.0)	26	275.5 (181.7, 313.7)	21	289.0 (167.0, 446.0)	0.240	0.200
Holo-TC (pM)	27	40.7 (23.3, 81.9)	26	79.4 (39.2, 125.0)	21	96.1 (39.4, 125.0)	0.021*	0.048*
Folate (nM)	27	55.9 (37.9, 70.8)	26	52.0 (36.8, 68.1)	21	42.7 (31.3, 80.0)	0.473	0.278
Vitamin B2 (pM)	26	357 (73.7)	25	316 (73.5)	20	314 (67.3)	0.053	0.924
Vitamin B6-pyridoxal-5-phospate (pM)	12	29.3 (18.6, 42.7)	13	25.0 (19.1, 39.5)	13	17.5 (11.6, 43.9)	0.494	0.298
Vitamin B6-pyridoxal (pM)	26	4.80 (3.6, 7.9)	25	5.70 (4.6, 8.3)	21	4.80 (3.2, 6.9)	0.187	0.806
Homocysteine (μmol/L)	27	8.30 (6.8, 11.6)	26	6.30 (4.8, 9.8)	21	6.60 (4.6, 11.9)	0.134	0.342
BDNF (pg/ml)	27	70.0 (31.0, 299.0)	26	106.0 (31.0, 412.2)	21	195.0 (31.0, 512.0)	0.620	0.364

*Values represented as Mean (SD), Median (25th, 75th) or n (%)*.

*p < 0.05, **p < 0.01, ***p < 0.001 p-values calculated by t-test.

*IQ, Intelligence Quotient; Holo-TC, holotranscobalamin; BDNF, Brain Derived Neurotrophic Factor; MMN, multi micronutrient*.

# *The number of women attending the follow up differed across the three time points*.

Hemogram was measured on a Beckman Coulter analyzer (AC.T diffTM Analyzer, Florida, USA) on the day of the collection. Plasma vitamin B12 and folate were measured using a microbiological assay and total homocysteine, vitamin B2 and B6 by HPLC (PerkinElmer 200 Series, PerkinElmer, Shelton, CT, USA). Plasma holo-TC was measured by a two-step immunoassay using CMIA technology (Architect, Abbott GmbH & Co. KG, Germany). This represents the fraction of vitamin B12 transported on transcobalamin-II and is available for the peripheral tissues, hence also called “active” vitamin B12. It is increasingly used as a more sensitive marker for B12 deficiency. The remaining vitamin B12 (70–80%) is attached to haptocorrin and is not available for peripheral tissues. Total vitamin B12 (called vitamin B12) is the sum of the two. Plasma BDNF was measured in cord blood using ELISA kit (XpressBio, Frederick, USA).

### Neurodevelopmental Assessments

The offspring born in the trial were followed up every 6 months until 2 years of age for measurements of their growth. Once they reached 24 months of age, the parents were approached regarding participation in the neurodevelopment study, and their written informed consent was obtained. The neurodevelopmental assessment was performed at the Child Development Center (TDH center), KEM Hospital, Pune.

The neurodevelopmental assessment was performed using the Bayley's Scale of Infant Development (BSID-III) (27). The BSID-III assesses the developmental status of infants from 1 to 42 months of age. The scales assess five domains across three main subscales: (1) cognitive (2) language—receptive and expressive language and (3) motor—which assesses gross and fine motor skills. The assessment was performed by trained clinical psychologists certified to perform the BSID-III. Testing was carried out in a quiet room, with a parent or guardian present, and instructions were provided in a language that was comfortable for the child. All children were assessed between 24 and 42 months of age. Each test protocol was independently reviewed and scored by two raters. The BSID-III test yields raw scores based on the performance of the child on test items for cognitive, expressive, and receptive communication, and fine and gross motor skills. The raw scores were converted into age standardized scaled scores as recommended in the manual. Summation of the scaled scores yields 3 composite scores for the cognitive, language and motor skills domains. We used the composite scores in our analysis. Composite scores were categorized into average, below or above average performance, based on standardized criteria provided in the manual, where the average is 100 with SD of 15 and a score of <85 is considered to be below average (27).

As part of ongoing assessments in the PMNS cohort, maternal intelligence [determined by the mothers' Intelligence Quotient (IQ) score] was assessed in some of the mothers at age 22–24 years using the Weschler's Adult Intelligence Scale-IV (WAIS-IV).

### Ethical Considerations

Details of community participation in the planning of this trial have been described earlier (26). The original PRIYA trial was approved by the KEM Hospital Research Centre Ethics committee and monitored by a Data Safety Monitoring Board (DSMB) and a Scientific Advisory Committee (SAC). The trial was registered with the CTRI (2012/12/003212) and ISRCTN (32921044). Neurodevelopmental follow up of the offspring was approved by the KEM Hospital Research Centre Ethics committee and registered in (clinical trials.gov ID: NCT03088189). Written informed consent was obtained from the parents of the children before conducting the neurodevelopmental assessment.

### Statistical Analysis

The purpose of our analysis was to see if pre-conceptional B12 and micronutrient supplementation in the mothers led to improvement in offspring neurodevelopmental performance (composite BSID-III scores) at 2 years of age. We also investigated the effect of supplementation on circulating vitamin levels in the mother and cord blood, and on cord blood BDNF levels.

We first examined whether randomization had equally distributed potential confounders such as parental education and standard of living index, maternal age, IQ, and anthropometry, length of supplementation and compliance across the three supplementation groups.

All data were represented as either mean and standard deviation (for normally distributed variables) or median and 25–75th percentile (for skewed variables). The skewed outcome variables (maternal and child biochemical measures, birth outcomes and neurodevelopmental measures) were log transformed. We used Pearson's correlation coefficient to test associations between the length of supplementation and biochemical measures at 28-week gestation and offspring cord blood. We compared differences in outcome variables between B12 alone or B12+MMN groups and the placebo group using the *t*-test. Adjustments for additional covariates (e.g., maternal B12 levels at screening) were performed using ANCOVA. We also examined longitudinal changes in the logarithmic values of vitamin B12 concentrations between time points and treatment groups using a two-way repeated measures ANOVA. Further, we examined the additional effect of duration of supplementation and compliance in the same model. We used a non-parametric test (Mann-Whitney *U*-test) to test the significance of difference in cord BDNF values between the supplementation groups because BDNF values could not be normalized by various transformations. Statistical analysis was performed using SPSS 25.0 and R statistical software 4.1.1.

## Results

Of the 266 women randomized in the trial, 182 were married, 166 became pregnant, and 149 delivered a live baby (Figure 1). Between May 2017 and February 2020, we approached the parents of 85 children who had attained the age of 2 years, for participation in the neurodevelopmental study. We had to halt the assessments after February 2020 due to the COVID-19 pandemic. None of the children had significant neurodevelopmental disorders (cerebral palsy, seizure disorders, or neural tube defects). Seven children who were above the inclusion age of 42 months as per the BSID norms, were excluded from analysis after confirming that they had achieved appropriate neurodevelopment for 42 months of age. Assessment could not be completed in 4 children. Our analysis is based on the remaining 74 children. The median age of the children at the time of performing the BSID was 29 months (Table 1). There were 42 boys and 32 girls; of these, 27 were in the placebo group, 26 in the B12 + MMN and 21 in the B12 alone group. There were no differences in gestational age at delivery, birth weight, length or head circumference amongst the offspring in the three supplementation groups. Similarly, there were no differences in parental education, standard of living index, maternal age, or IQ (Table 1).

The children who were not invited for the study because they were below 24 months of age differed from those studied; they had higher socio-economic status and parental education, higher maternal and cord B12 and holo-TC, and lower cord homocysteine compared to the study group (Supplementary Table 2).

### Effect of Supplementation on Maternal and Newborn Micronutrient Status, and Birth Measures

At baseline, maternal B12 and holo-TC levels were similar across the three supplementation groups (Table 1). Fifty one percent of the participants had vitamin B12 deficiency at screening (B12 <150 pM), and this reduced to 22% at 6–12 months after starting supplementation. There was a rise in vitamin B12 and holo-TC levels in the B12 supplemented groups compared to the placebo group, both pre-conceptionally (18 years of age) and at 28 weeks of gestation. There was no significant association between length of supplementation (from start of supplementation till date of 28 weeks gestation and date of delivery) and circulating concentration of vitamin B12 in either group. There was a significant association between length of supplementation and holo-TC concentrations in the cord blood of the B12 alone group (*r* = 0.462 *p* = 0.035).

Repeated measures ANOVA showed a significant effect of time (*F* = 18.517, *p* < 0.001) and treatment (*F* = 9.363, *p* < 0.001) on log serial B12 concentrations. Addition of length of supplementation and compliance did not change the result. *Post hoc* comparisons using Bonferroni correction, showed that the log B12 concentrations in both B12 + MMN (95% CI = 0.14, 0.56, *p* < 0.001) and B12 alone (95% CI = 0.17, 0.63, *p* < 0.001) groups were significantly higher than the placebo group. There was no difference in vitamin B12 concentrations between B12 + MMN and B12 alone groups. Cord blood levels of holo-TC were significantly higher in both the B12 supplemented groups compared to the placebo group, though vitamin B12 levels were similar.

Baseline plasma homocysteine concentrations were high but similar in the three supplementation groups, and fell substantially in the vitamin B12 supplemented groups pre-conceptionally. During pregnancy, as expected, plasma homocysteine concentrations fell in all groups. They were similar in the three groups during pregnancy and in the cord blood.

Circulating folate concentrations were similar at baseline in the three groups and increased during pregnancy (due to supplementation). Folate levels were significantly lower at 28 weeks gestation in the B12 + MMN group compared to those in the placebo group. Folate levels were similar in the cord blood across the groups. Circulating B2 levels were higher in the B12 + MMN group as compared to the placebo group during 28 weeks gestation.

Hemoglobin concentrations were similar in the mother and the offspring across all the groups.

### Comparison of BSID Scores and Cord BDNF Between Supplementation Groups

Age standardized composite scores for the domains of cognition, motor and language development were obtained on 74 children. There was no difference in performance between males and females (Supplementary Table 3). No significant developmental delays were observed in any of the children (score <69). Few children showed a below average performance on the cognitive (4.1%, *n* = 3), motor (4.2%, *n* = 3), and language domain (8.3%, *n* = 6) (score <85) (Supplementary Table 4).

**Figure 2:**
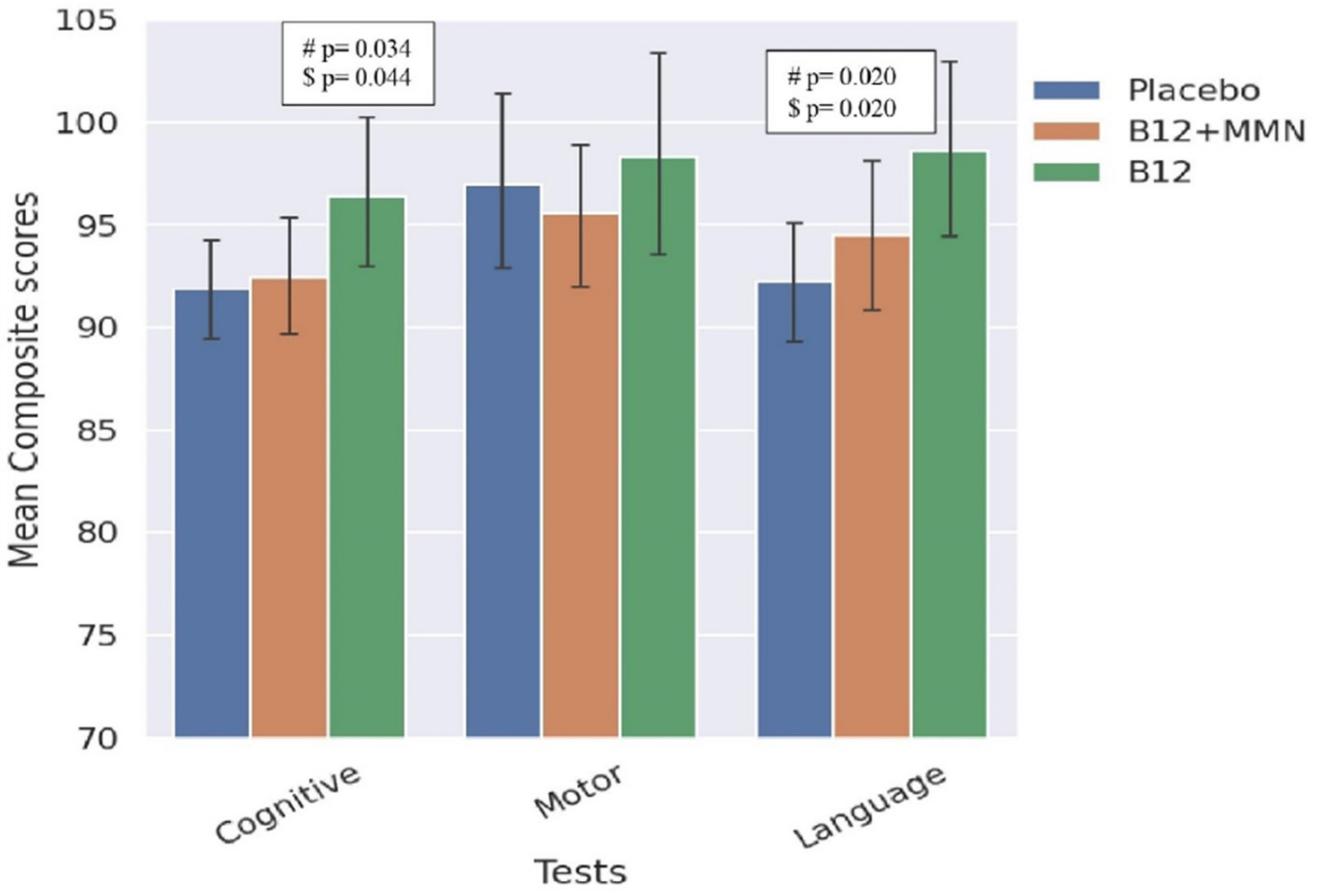
Bar graph comparing scores in BSID-III domains between treatment and placebo groups. Comparison between placebo and treatment groups on BSID-III domains. ^#^significance at *p* < 0.05, ^*$*^significance *p* < 0.05 value adjusted for maternal baseline B12 levels. Error bars represent 95% confidence intervals.

The offspring of mothers in the B12 alone group performed the best in the cognitive and language domains, and significantly better than the placebo group (Table 2, Figure 2). This difference persisted after adjusting for the baseline plasma vitamin B12 concentrations. Cognition and language composite scores were 5–7% higher in the B12 alone group than the placebo group.

**Table 2:** Comparison between placebo and supplemented groups in BSID-III domains.

**BSID-III domains**	**Placebo group**	**B12 + MMN group**	**B12 group**	**^#^** ***p*** **-value**		**^$^** ***p*** **-value**	
				**Group (B12 + MMN vs. Placebo)**	**Group (B12 vs. Placebo)**	**Group (B12 + MMN vs. Placebo)**	**Group (B12 vs. Placebo)**
Cognitive	90.0 (85.0, 95.0)	90.0 (85.0, 96.2)	95.0 (90.0, 100)	0.969	0.034^*^	0.781	0.044^*^
Motor	94.0 (91.0, 100.0)	95.5 (90.2, 100.0)	97.0 (91.0, 107.0)	0.687	0.818	0.522	0.384
Language	92.2 (7.8)	93.7 (9.87)	98.6 (10.1)	0.556	0.020^*^	0.633	0.020^*^

*Values represented as mean (SD) or Median (25th, 75th)*.

* *p < 0.05*.

# *P-value calculated by t-test*.

$ *P-value calculated by ANCOVA; adjusted for maternal baseline B12 levels*.

*Language performance was normally distributed, and mean (SD) are reported. MMN, multi micronutrient*.

There were no significant differences between the B12 + MMN group and the placebo group on any of the neurodevelopmental domains.

The two supplementation groups had higher cord BDNF values than the placebo group, the B12 alone group had the highest values, however the difference was non-significant (Table 1). Cord blood BDNF values did not show significant associations with any of the BSID-III composite scores.

## Discussion

In this rural Indian population with a substantial prevalence of B12 deficiency, we found that supplementation of adolescents with 2 μg/day of B12 significantly improved their own B12 status (total B12 and holo-TC) and offspring cord blood holo-TC. Offspring whose mothers received vitamin B12 alone performed better than offspring of mothers in the placebo group in neurodevelopmental assessments (cognitive and language domain of the BSID-III test at 24–42 months of age). Offspring whose mothers received B12 + MMN performed similarly to the placebo group.

The role of pre-conceptional folic acid supplementation in preventing NTDs is well-established, especially in western (mainly non-vegetarian) populations (23, 24). In vegetarian populations like India, vitamin B12 is likely to play a similar role, because both folate and B12 act as cofactors for the enzyme methionine synthase, in methylation reactions. Studies in India have highlighted an association of both maternal vitamin B12 and folate with different outcomes in the offspring including neurodevelopmental performance. Studies in Pune showed an association of low maternal vitamin B12 status (low holo-TC concentrations and TCN2 polymorphisms) with an increased risk of NTD, and a positive association between maternal vitamin B12 status during pregnancy and offspring neurocognitive performance at 2 and 9 years of age (5, 18, 19). A study in North Indian children aged 12–18 months found that both vitamin B12 and folate status had significant associations with cognitive performance (28) while a study in Mysore found that higher maternal folate concentrations, but not vitamin B12, during pregnancy were associated with better cognitive ability in children at 9–10 years of age (29). Adequate status of both vitamins is likely to be important for brain development and function. Recent systematic reviews, including both observational and interventional studies, provide a moderate level of evidence for a role of maternal B12 status in determining offspring cognitive function, and highlight a need for more studies from developing countries (17, 30). Studies in Mexico and Singapore have also reported an association between maternal dietary intake of vitamin B12 and offspring cognitive abilities (31, 32). Observations in the ALSPAC cohort in the UK suggests a weak association of a maternal genetic determinant of circulating vitamin B12 concentrations (*FUT*2) and offspring IQ at 8 years of age (33). On the other hand, a cohort study in Canada showed no significant associations between maternal vitamin B12 concentrations and BSID-III outcomes in their offspring at 18 months (32). This may be due to a lack of significant variation in maternal vitamin B12 status, given the low prevalence of vitamin B12 deficiency in their population (34).

Our findings from this pre-conceptional maternal micronutrient supplementation trial fills an important gap in the literature. Our observations are supported by a maternal B12 supplementation study from south India, which supplemented mothers with 50 μg vitamin B12 from the 1st trimester of pregnancy until 6 weeks postpartum. Supplementation improved maternal B12 levels in the third trimester (20) and offspring had better neurodevelopmental scores (language domain) at 30 months of age (21). In another trial, vitamin B12 (1.8 μg) and/or folic acid (150 μg) supplementation in 6–30-month-old children for a period of 6 months showed improvement in their neurocognitive performance (35). In this study B12 alone group showed improvement in gross motor functioning and the B12 + folic acid group in gross motor as well as problem-solving functioning compared to the placebo; folic acid alone had no effect.

A high prevalence of B12 deficiency is unique to the Indian context due to the socio-cultural practice of vegetarianism. In a previously published systematic review (17), we found a high prevalence of B12 deficiency during pregnancy reported from southern (51%), western and northern India (70–74%). Severe absorption defects (i.e., pernicious anemia) are rare and vitamin B12 deficiency is largely a low dietary intake problem (15–18). This offers a unique opportunity to control a modifiable risk factor at the public health scale to improve neurodevelopment and human capital in the next generation. The utility of this approach in populations with a high consumption of fish and meat (e.g., in coastal areas of India) will need to be examined, keeping in mind that folate may be more important than B12 in these populations. Our choice of a near- recommended dietary allowance (RDA) dose of B12 (2 μg/day) was based on our earlier studies showing adequate absorption of oral B-12 (36) in this population and the demonstration in a pilot study of improvement in B-12 and homocysteine status after oral supplementation with 2 or 10 μg/day for 1 year (37). In another study of severely B12 deficient girls (plasma B12 <100 pmol/l), we demonstrated an improvement in hematological parameters and peripheral and autonomic nerve functions after supplementing 2 μg/day of vitamin B12 for 11 months (38). In the present study we found a rise in both total B12 and holo-TC levels in the supplemented groups within a few months of starting supplementation at 28 weeks gestation, and in the cord blood. Use of a small dose of vitamin B12 makes our results important for public health actions. Thus, we believe that our current study fills an important gap to help public health policy to include supplementation with a physiological dose of vitamin B12 among adolescents and reproductive age women. This may improve not only their own health, but that of the next generation's as well. Being aware of the difficulties of achieving long term compliance with tablet supplementation in relatively asymptomatic individuals, we have recently reported the efficacy of commonly eaten vitamin fortified food items (a nutrient bar and yogurt) to achieve better vitamin B12 status (39). All these approaches are usable in the national programmes to improve micronutrient nutrition of children, adolescents and pregnant mothers. The improved cognitive outcomes were seen specifically in the B12 alone supplemented group and not in the B12 + MMN group. We are unsure about the reasons for this. Though the circulating levels of vitamin B12, holo-TC, and BDNF appeared higher in the B12 alone group, the difference from the B12 + MMN group was not significant. There was no difference in the compliance and length of supplementation in different groups. Maternal IQ, parental education, and socio-economic status which may influence child neurodevelopment were also similar. It has been postulated that administration of a combination of multiple micro nutrients may interfere with actions of each other (40), such a mechanism could operate in the B12 + MMN group. It is notable that the effects of maternal multiple micronutrient supplementation on offspring outcomes are inconsistent. A systematic review from 9 trials (6 of which used the UNIMMAP micronutrient formulation) did not find favorable effects on child mortality, birth size, or offspring cognition (41).

Vitamins B12 and folate participate in the one-carbon metabolism pathway to stimulate synthesis of precursor nucleotides for DNA synthesis, and also generate the universal methyl donor S-Adenosyl methionine (SAM) which is involved in methylation of DNA (an important epigenetic mechanism), proteins and lipids and generating neurotransmitters (42, 43). These mechanisms are reputedly involved in fetal growth and differentiation and a deficiency or imbalance of these vitamins may result in a permanent change in the structure and function of developing tissues which may manifest as disorders in later life (“fetal programming”) (1). We have demonstrated alterations in adiposity and insulin resistance in children whose mothers had an imbalance of these vitamins (low B12—high folate) during pregnancy (11). Animal studies have shown differences in the expression of neurotrophic factors such as BDNF in the brains of fetuses whose mothers were exposed to low vitamin B12 status (9). In our study, though we did not find significant differences in cord blood BDNF concentrations between supplementation groups, the values tended to be higher in the B12 alone group. Further studies are required to understand the utility of cord BDNF levels as a neurodevelopmental marker in humans.

Neurodevelopment is a dynamic process that involves neurogenesis, neuronal migration, cortical growth and gyrification, starting in early pregnancy and lasting until infancy (first 1,000 days). The pre- and periconceptional period is an important window within this broader window because of “epigenetic reprogramming” of the conceptus which happens within 48–72 h of conception (44). The majority of pregnancies are unplanned, and women approach the healthcare system after this window. Our supplementation was specifically started in adolescence to ensure adequate micronutrient stores in the mother from before conception, in time to support gametogenesis, conception, embryogenesis, organogenesis, and placentation (43, 45). The success of pre-conceptional folic acid supplementation in preventing NTDs is well-known (23–25). Thus, we propose that the 1,000-day window should be expanded to include the preconception period. This would shift the action from the clinic to the community and will fit well into a multitude of adolescent and reproductive age programs across the world.

Additional strengths of the PRIYA is that it is a trial within a cohort in which original observations were made. The randomized controlled trial design ensured that potential confounders were similarly distributed between allocation groups. High rates of participation in the trial, high rates of follow up, and of sample collection at delivery are also noteworthy. Exclusion of women with severe B12 deficiency (<100 pM) from a placebo-controlled trial on ethical grounds reduced the power of the study because they and their offspring could have benefited the most from the B12 supplementation. The COVID pandemic also interfered with our ability to test more children for neurodevelopment and meant that we missed the children of women who became pregnant later. Despite these limitations we were able to see the beneficial effects of the supplementation. We expect that the performance on the Bayley's scale will reflect in neurodevelopmental indices at a later age. This will be tested during subsequent follow ups.

## Summary and Conclusion

We found that pre-conceptional maternal supplementation with a near RDA dose (2 μg/day) of vitamin B12 exposed their offspring to higher vitamin B12 status peri-conceptionally and during pregnancy. This was associated with better neurodevelopmental performance in the children, in cognitive and language domains, between 24 and 42 months of age. Our study highlights an important role for maternal vitamin B12 in offspring neurodevelopment. We urge that the first 1,000 days window be extended to include the pre-conceptional period. Our findings have strong implications for public health policy to improve the vitamin B12 status of young adolescents and reproductive age women in populations with a sizable prevalence of vitamin B12 deficiency. Utility of this approach in non-vegetarian populations, needs to be documented. We foresee benefits of such a policy to many national nutrition programmes in India.

## Data Availability Statement

The raw data supporting the conclusions of this article will be made available by the authors, without undue reservation.

## Ethics Statement

The studies involving human participants were reviewed and approved by KEM Hospital Research Center Ethics Committee. The patients/participants provided their written informed consent to participate in this study.

## Author Contributions

CSY and RVB designed the neurocognitive follow-up study. ND and RVB analyzed the data and wrote the first draft. BP and MD performed and reported the neurocognitive assessments. DB performed the biochemical measurements. SB and AB contributed to the statistical analysis. SS and RS conducted the follow-up of the participants. KK, RL, and PY contributed to conducting the PRIYA trial. CSY and CF designed the original PRIYA trial. CSY and CF edited the final manuscript. All authors contributed to the article and approved the submitted version.

## Funding

The PRIYA trial is funded by the Indian Council of Medical Research (58/1/8/MRC-ICMR/2009/NCD-II) and Medical Research Council, UK (MR/J000094/1) as part of an Indo-UK collaborative call. The biological sample collection and analysis was funded by the DBT-CEIB grant BT/PR12629/MED/97/364/2016. The neurocognitive follow up and cord BDNF measurements was supported by the DBT/Wellcome India Alliance Fellowship (IA/CPHI/161502665) awarded to RB. CY was visiting professor at Danish Diabetes Academy & Southern University of Denmark (2016–2018) which was funded by NOVO-NORDISK FONDEN.

## Conflict of Interest

SKB was employed by Cytel Inc. The remaining authors declare that the research was conducted in the absence of any commercial or financial relationships that could be construed as a potential conflict of interest.

## Publisher's Note

All claims expressed in this article are solely those of the authors and do not necessarily represent those of their affiliated organizations, or those of the publisher, the editors and the reviewers. Any product that may be evaluated in this article, or claim that may be made by its manufacturer, is not guaranteed or endorsed by the publisher.
