# Corrigendum: Pre-conceptional Maternal Vitamin B12 Supplementation Improves Offspring Neurodevelopment at 2 Years of Age: PRIYA Trial

**DOI:** 10.3389/fped.2022.860732

**Published:** 2022-02-21

**Authors:** Naomi D'souza, Rishikesh V. Behere, Bindu Patni, Madhavi Deshpande, Dattatray Bhat, Aboli Bhalerao, Swapnali Sonawane, Rohan Shah, Rasika Ladkat, Pallavi Yajnik, Souvik K. Bandyopadhyay, Kalyanaraman Kumaran, Caroline Fall, Chittaranjan S. Yajnik

**Affiliations:** ^1^Diabetes Unit, King Edward Memorial Hospital Research Center, Pune, India; ^2^Terre des Hommes Rehabilitation and Morris Child Development Centre at KEM Hospital, Pune, India; ^3^Strategic Consulting, Cytel Inc., Cambridge, MA, United States; ^4^Medical Research Council Lifecourse Epidemiology Unit, University of Southampton, Southampton, United Kingdom

**Keywords:** vitamin B12, pre-conception, supplementation, neurodevelopmental outcome, offspring

In the original article, there was a an error in the number of females given in the **Abstract**, sub-section ***Methods***. The sentence “the Pune Rural Intervention in Young Adolescents trial (PRIYA), adolescents (*N* = 557, 226 females)” should instead read “the Pune Rural Intervention in Young Adolescents trial (PRIYA), adolescents (*N* = 557, 266 females).”

The corrected sub-section appears below:

**Methods:** In the Pune Rural Intervention in Young Adolescents trial (PRIYA), adolescents (*N* = 557, 266 females) were provided with vitamin B12 (2 μg/day) with or without multiple micronutrients, or a placebo, from preconception until delivery. All groups received mandatory iron and folic acid. We used the Bayley's Scale of Infant Development (BSID-III) at 24–42 months of age to investigate effects on offspring neurodevelopment.

The authors apologize for this error and state that this does not change the scientific conclusions of the article in any way. The original article has been updated.

## Publisher's Note

All claims expressed in this article are solely those of the authors and do not necessarily represent those of their affiliated organizations, or those of the publisher, the editors and the reviewers. Any product that may be evaluated in this article, or claim that may be made by its manufacturer, is not guaranteed or endorsed by the publisher.

