# Supplementary material for: Pre-conceptional Maternal Vitamin B12 Supplementation Improves Offspring Neurodevelopment at 2 Years of Age: PRIYA Trial
Source: Front Pediatr. 2021 Dec 7;9:755977. doi: 10.3389/fped.2021.755977 (PMC8697851; doi:10.3389/fped.2021.755977)
Supplement: Supplementary file 1 [file Table_1.pdf]

Supplementary Table 1. Contents of the multiple micronutrient supplement used in the PRIYA trial

Contents	Dose
Vitamin A (μg)	300
Vitamin D (IU)	100
Vitamin E (mg)	5
Vitamin C (mg)	20
Vitamin B1 (mg)	0.75
Vitamin B2 (mg)	0.9
Vitamin B3 (mg)	10
Vitamin B6 (mg)	0.5
Vitamin B12 (μg)	1.0
Zinc (mg)	6
Copper (mg)	1
Selenium (μg)	20
Iodine (μg)	75

PRIYA trial provided 2 μg/day of Vitamin B12 to the B12 alone and the B12+MMN groups. To satisfy the local regulatory guidance, we provided it in 2 capsules, each containing 1 μg of Vitamin B12. Iron and Folic Acid were excluded from the UNIMMAP composition because it was separately provided in the IFA tablets (Iron and Folic Acid). The placebo group received only the excipients. Additional details are provided in the PRIYA methods paper (26).

Supplementary Table 2. Maternal and child characteristics of the assessed group compared to the non-assessed group

Variables	n	Assessed group	n	Not assessed group	P value
**Parental sociodemographic characteristics**					
Maternal age at 28 weeks gestation (years)	82	19.6 (18.5, 20.3)	62	22.5 (21.6, 23.0)	<0.001***
Maternal education (years)	84	12.0 (10.0, 13.0)	62	15.0 (12.0, 15.0)	<0.001***
Maternal height (cms)	82	157.0 (5.1)	61	156.0 (4.8)	0.067
Maternal weight at 28 weeks gestation (kgs)	82	52.1 (48.4, 57.7)	61	55.1 (50.1, 60.5)	0.915
Maternal IQ	58	75.5 (71.0, 83.0)	29	81.0 (75.5, 88.0)	0.023**
Standard of Living Index	84	36.5 (31.2, 40.0)	62	36.5 (31.0, 40.2)	0.763
Paternal Education (years)	84	12.0 (10.0, 15)	62	15 (12, 15)	0.006**
**Maternal Micronutrients**					
Maternal B12 at screening (pM)	85	148 (125, 202)	64	147 (127, 187)	0.677
Maternal Folate (nM) at screening	85	20.9 (13.8, 27.1)	64	18.5 (15.0, 25.3)	0.965
Maternal Homocysteine (µmol/L) at screening	85	21.1 (16.4, 32.4)	64	22.0 (17.9, 33.8)	0.416
Maternal B12 at 18 years (pM)	76	225 (162, 326)	61	226 (152, 287)	0.861
Maternal Folate (nM) at 18 years	78	21.2 (15.8, 29.7)	61	23.2 (18.1, 31.0)	0.130
Maternal Homocysteine (µmol/L) at 18 years	79	11.8 (9.30, 16.8)	61	11.5 (9.60, 18.7)	0.583
**Maternal Micronutrient levels at 28 weeks gestation**					
Hemoglobin (gm/dl)	82	10.4 (9.40, 11.0)	61	10.6 (9.80, 11.6)	0.078
B12 (pM)	82	166 (126, 235)	61	210 (156, 305)	0.005**
Holo-TC (pM)	82	19.6 (12.5, 31.0)	61	34.7 (20.0, 89.3)	<0.001**
Folate (nM)	82	31.4 (16.2, 61.1)	61	45.6 (24.0, 60.2)	0.122
B2 (pM)	81	243.0 (221.0, 275.5)	61	198.0 (175.5, 238.0)	<0.001***
B6-pyridoxal-5-phospate (pM)	81	3.80 (2.90, 5.50)	61	4.10 (2.90, 5.60)	0.572
B6-pyridoxal (pM)	81	1.20 (0.94, 1.60)	61	1.60 (1.20, 2.30)	<0.001***
Homocysteine (µmol/L)	82	6.60 (4.27, 8.20)	61	6.60 (5.20, 8.95)	0.281
**Child Characteristics**					
Gender	85	Boys= 48 (56.5%)	63	Boys= 31 (49.2%)	
**Birth Anthropometry**					
Birth weight (gms)	85	2700 (2377, 3000)	64	2780 (2545, 3015)	0.338
Birth length (cms)	84	48.2 (47.0, 49.2)	60	48.2 (47.1, 49.1)	0.943
Head circumference (cms)	84	33.1 (32.4, 33.9)	60	33.5 (32.8, 34.3)	0.020*
Gestation age (months)	85	39.2 (38.2, 40.1)	64	39.5 (38.7, 40.1)	0.562
**Cord Micronutrients**					
B12 (pM)	85	243 (165, 373)	58	369 (223, 808)	0.001**
Holo-TC (pM)	85	61.3 (27.5, 119)	40	121 (47.2, 128)	0.004**
Folate (nM)	85	9.30 (6.55, 15.5)	57	10.3 (7.45, 13.8)	0.730
B2 (pM)	81	321 (263, 379)	58	274 (242, 305)	<0.001***
B6-pyridoxal-5-phospate (pM)	85	26.0 (15.8, 37.6)	58	27.4 (17.2, 38.7)	0.557
B6-pyridoxal (pM)	85	4.90 (3.75, 7.15)	58	4.50 (3.47, 6.10)	0.138
Homocysteine (µmol/L)	85	7.40 (5.00, 10.7)	58	6.20 (4.25, 8.20)	0.020*

*p<0.05, **p<0.01, ***p<0.001 p-values calculated by students t-test

Values represented as Mean (SD) Median (25th, 75th) or n (%)

IQ, Intelligence Quotient; Holo-TC, holotranscobalamin; BDNF, Brain Derived Neurotrophic Factor; MMN, multi micronutrient

Supplementary Table 3. Comparison of BSID performance between the sexes

BSID-III domains	Male	Female	p value
Cognitive	90.0 (85.0, 95.0)	95.0 (90.0, 100)	0.099
Motor	94.0 (89.5, 100)	97 (91.0, 107)	0.329
Language	93.2 (10.4)	96.4 (7.71)	0.160

Values represented as Mean (SD) or Median (Q1, Q3)

p-values calculated by t-test

Supplementary Table 4. Comparison of BSID performance categories across treatment groups

**BSID-III domains**	**Category**	**Placebo**	**B12+MMN**	**B12**	**Chi-square p value**
Cognitive	Above average	0%	3.8%	9.5%	0.480
	Average	96.3%	92.3%	90.5%	
	Below average	3.7%	3.8%	0%	
Motor	Above average	8.0%	11.5%	9.5%	0.904
	Average	88.0%	84.6%	90.5%	
	Below average	4.0%	3.8%	0%	
Language	Above average	0%	3.8%	15.0%	0.241
	Average	96.2%	88.5%	80.0%	
	Below average	3.8%	7.7%	5.0%	

Values represented as Mean (SD) or Median (Q1, Q3)

p-values calculated by chi-square

MMN, multi micronutrient
